# The Anther Steps onto the Stigma for Self-Fertilization in a Slipper Orchid

**DOI:** 10.1371/journal.pone.0037478

**Published:** 2012-05-23

**Authors:** Li-Jun Chen, Ke-Wei Liu, Xin-Ju Xiao, Wen-Chieh Tsai, Yu-Yun Hsiao, Jie Huang, Zhong-Jian Liu

**Affiliations:** 1 Shenzhen Key Laboratory for Orchid Conservation and Utilization, The National Orchid Conservation Center of China and The Orchid Conservation & Research Center of Shenzhen, Shenzhen, China; 2 The Center for Biotechnology and BioMedicine, Graduate School at Shenzhen, Tsinghua University, Shenzhen, China; 3 Continuing Education College of Beijing Forestry University, Beijing, China; 4 Institute of Tropical Plant Sciences, and Orchid Research Center, National Cheng Kung University, Tainan, Taiwan, China; 5 Department of Life Sciences, National Cheng Kung University, Tainan, Taiwan, China; 6 College of Forestry, South China Agricultural University, Guangzhou, China; Montreal Botanical Garden, Canada

## Abstract

**Background:**

Due to the spatial separation between male and female pollen grains from the anther of most flowering plants, including orchids, pollens are transported by wind or animals and deposited onto the receptive surface of the stigma of a different plant. However, self-pollination is common in pollinating animal-scarce habitats. In such habitats, self-pollinations require the assistance of a pollinating agent (e.g., wind, gravity, or floral assembly) to transport the pollen grains from the anther onto its own stigma.

**Methodology/Principal Findings:**

Based on observations on floral morphology and flowering phenology, tests of the breeding system, and a comparison of pollination mechanisms, a new self-pollination process was discovered in the hermaphroditic (i.e., possessing spatially separated male and female organs) flower of a slipper orchid, *Paphiopedilum parishii*. The anther changes from a solid to a liquid state and directly steps onto the stigma surface without the aid of any pollinating agent or floral assembly.

**Conclusions:**

The mode of self-pollination discussed here is a new addition to the broad range of genetic and morphological mechanisms that have evolved in flowering plants to ensure their reproductive success. The present self-contained pollination mechanism is a possible adaptation to the insect-scarce habitat of the orchid.

## Introduction

Reproduction plays a key role in the process of plant evolution. The transformation of the reproductive system from outcrossing to selfing is the most common change in the evolution of angiosperms [1]. Owing to the frequent occurrence of inbreeding depression, plants have evolved numerous mechanisms to avoid selfing [2]. This phenomenon has resulted in promoting the space–time separation of male and female functions (e.g., dioecy, dichogamy, herkogamy, enantiostyly, and heterostyly) [3]. The purposes of this separation are to avoid the disturbance between pollen exportation (male function) and reception (female function), and achieve the accuracy of pollen transmission to reduce pollen waste [1], [4]. However, present-day plants have a high ratio of selfing. Darwin considered the assurance of reproduction to be the most important factor [5] that enables plants to develop selfing mechanisms. In environments where the external pollen is inadequate (e.g., due to a very low population density and few pollinators) and results in a failure of outcrossing, selfing is selected to ensure the fertilization of ovules or to obtain reproductive assurance. However, this process may cause a marked and rapid decrease in inbreeding [6]–[8].

Due to the spatial separation between male and female organs, pollen grains from the anther of most flowering plants are transported by wind or animals and deposited onto the receptive surface of the stigma of a different plant [9], [10]. In animal-pollinated flowers, floral structures ensure that pollen grains adhere to the pollinating agent; placing pollen on their own stigma during the period of receptivity is usually impossible. Consequently, the probability of facultative autogamy is ruled out, but self-pollination is also common. Most self-pollination processes require the assistance of a pollinating agent, e.g., wind or gravity [9], [10] and even animals [11], to transport pollen grains from the anther to its own stigma (passive self-pollination) [9]–[11]. Only a few species can overcome this obstacle in the floral structure. Such species move their floral components to achieve self-pollination in the late flowering phase (active self-pollination). For example, stigma lobes bend toward a pollen-transferred surface, stamens bend toward a stigma [12], [13], or pollens slip toward the stigma with the aid of excreted oil from the anther and a specific groove on the stigma surface [10], even selfing in the closed flower [13], [14].

To avoid selfing, orchids usually separate the anther and stigma by a rostellum [15]. In some species such as *Paphiopedilum*, the anther even grows far from the stigma [16], [17]. The labellum has also evolved to facilitate cross-pollination by insects. Thus, orchids generally adopt a strategy of cross-pollination, or in the case of lacking pollinators, apply a mixed strategy of cross- and self-pollination [18] or autonomous self-pollination [19]. Brown [19] first observed that the floral structure of *Ophrys apifera* is adapted for self-fertilization. The anther caps naturally open soon after the flower of *O. apifera* is fully expanded, and the pollinium falls from the anther onto its own stigma by the aid of gravity and wind [19]. The review of Catling [13] on the incidence of autonomous self-pollination in orchids shows that autonomous self-pollination occurs in all orchid subfamilies. Van der Cingel [20], [21] provides a more complete estimate of the importance of autonomous self-pollination in Orchidaceae [22], suggesting that autonomous self-pollination is common in this family. Orchids have a variety of autonomous self-pollination mechanisms. For example, Catling [14] reported some cases of movement of anther or pollinia in which friable pollen or a whole anther falls onto the stigma surface [13]. However, most need the aid of a pollinating agent and a few pollination modes need the aid of floral assembly. Commonly, the stigma is dilated on both sides and encircles the pollinium gradually, making it fall into its own cavity [23]. In habitats that lack a pollinating agent, some species develop a structure that links with pistils and stamens to assist the pollen in reaching the stigma and achieving self-pollination. For example, pollinia can enter the stigma cavity via the auto-spinning of the stipe. This type of autonomous self-pollination has been discovered in a recent study on the breeding system of *Holcoglossum* (now *Paraholcoglossum* [24]) *amesianum*. In the wild, the pollinia of this species can rotate 360° via the auto-spinning of the stipe. As a result, the pollinia stuff themselves into the stigma cavity of the same flower [9]. Another recent study on the pollination mechanism of *Jumellea stenophylla* revealed that there is no conspicuous spatial separation between the anther and stigma in its flowers. Pollinia increase in size (vacuolization) to come in contact with its own stigma cavity [25]. In the South African species *Eulophia*, the partial or complete absence of rostellum tissue allows contact between pollinia and stigmatic fluid. Thus, pollen tubes can grow from *in situ* pollinia to ovules [26]. In some races of slipper orchid, the style (stigmatic branch) is shortened (e.g., *Cypripedium passerinum*), the stigma is developed (e.g., *Phragmipedium lindenii*), or the filaments are bent (e.g., *Paphiopedilum lowii* and *Paphiopedilum. mastersianum*) for the stigma to touch the anther, resulting in selfing [13]. These self-pollination modes need the aid of movement or the absence of some floral assembly.

The present paper describes a newly discovered self-pollination process in the slipper orchid *Paphiopedilum parishii* [16], [17]. In this process, the solid-state anther can liquefy automatically, and the liquefied anther can step onto the receptive surface of the stigma through the distance between the anther and stigma. This self-pollination mechanism, which is not aided by any pollinating agent or medium, is a newly observed mode of reproduction in flowering plants.

## Results

**Figure 1:**
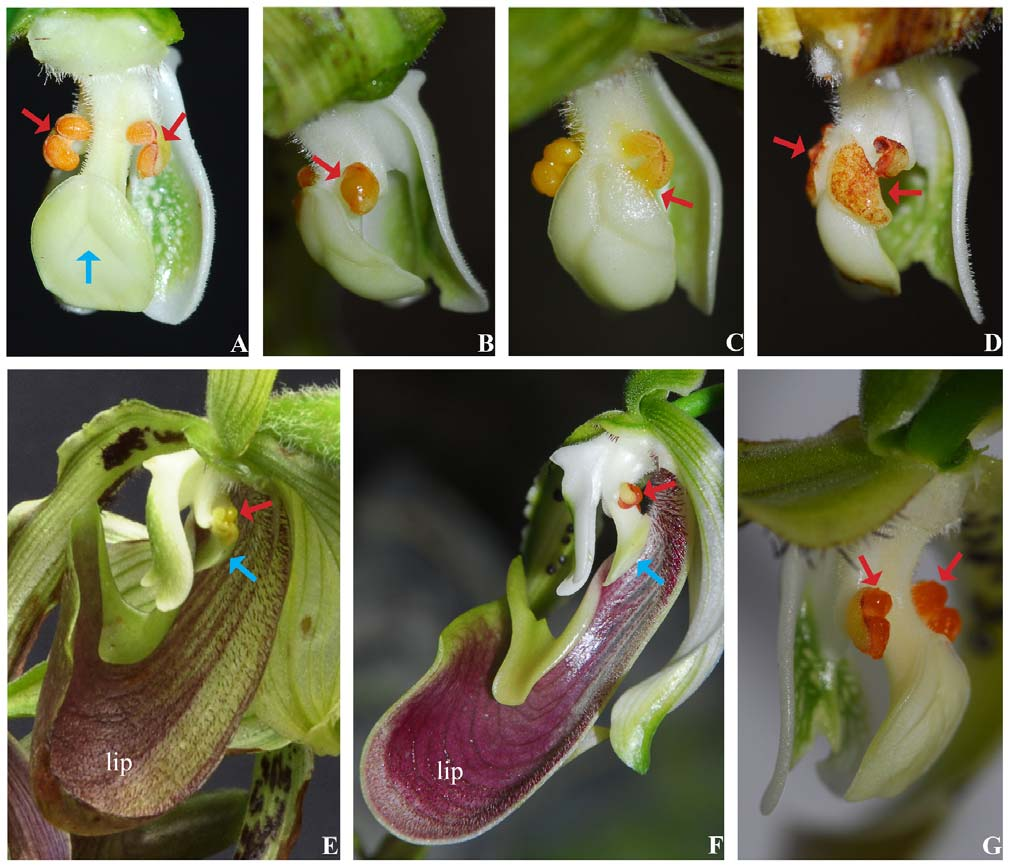
Pollination mechanism of *P. parishii*. (***A***) Structural characteristics of the column. The distance between the anther (red arrow) and stigma (blue arrow) is 2 mm to 3 mm (Figures 1B and 1E). (***B***) The flower moves backward and upward, making the both of anthers and the lateral lobes of the stigma lie in a horizontal plane, and solid-state pollinia begin to liquefy (red arrow). (***C***) The anther continues to liquefy close to the stigma and touches its margin (red arrow). (***D***) The anther liquid spreads onto the receptive surface of the stigma (red arrow). (***E***) Floral morphology and structure. The anther and stigma are shown in red and blue arrows, respectively. (***F***) Floral morphology and structure of *P. dianthum* pollinated by hoverflies. The anther and stigma are shown in red and blue arrows, respectively. (***G***) The column of *P. dianthum*, whose anthers do not liquefy (red arrow).

*P. parishii* grows on tree trunks or rocks in broad-leaved forests. This species flowers from July to September during the rainy season. The plants have one to four inflorescences that are 35–60 cm long, each with six to eight pale yellow-green flowers. The flowers are 8–10 cm across with their perianths dropping off, possessing the typical structure of the genus. Usually, there is only one flower on an inflorescence that opens every 3 days and lasts for 5–7 days. Following successful pollination, the corolla turns pale and wilts but remains on the young fruit. Its labellum, which is a pouch that acts as a trap for pollinators, is equipped with incurved margins to prevent the direct escape of trapped insects. At the bottom of the flower, there is a ladder of hairs leading up to the base of the labellum. This ladder is used by insects for climbing to escape through a gap on either side of the column, passing beneath the stigma and anther. The stigma is oval, trilobed, approximately 8 mm to 9 mm long and 4 mm to 5 mm wide. It has a downward receptive surface for pollen grains, and lies anterior to the basal channel of the labellum. There are two stamens, each with 3 mm to 4 mm filaments, situated near the exit of the basal channel side 2 mm to 3 mm away from the stigma. The anthers are long-ellipsoid and approximately 2 mm×1 mm in size (Figure 1A). This structure (Figure 1E) is considered typical for insect cross-pollination [16], [17]. However, after over 15 days (approximately 161 h) of observation, no insect that could have touched the anthers and stigmas visited the flowers, i.e., no insect was seen near or on the orchids. All 300 observed flowers did not secrete any nectar or odor, and 261 flowers set fruit. When the flower of this species was about to open, the entire anther began to liquefy and became pasty. The spreading-forward flower moved backward and upward. This movement adjusted the location of the anthers to the inclined top of the stigma when the flower fully opened from its original location (Figure 1B). The liquefied anther, which formed a liquid droplet, continuously liquefied and amplified. It then gradually moved close to either lateral lobe of the stigma (Figure 1C). As soon as the anther droplet made contact with the lobe edge, it stepped onto the papillate surface and rapidly spread onto the entire surface of stigma lobes (Figure 1D).

The liquefied anther spread rapidly. The liquefaction of the anther required a period of about 24 hours, and the fully liquefied anther took about 5 min to step onto the stigma. The spreading rate of plants in the 1aboratory at 80% to 95% relative humidity was comparable with that of plants in their natural habitats or forests with nearly saturated humidity. When the liquefied anther touched the stigma-lobe margin, it promptly covered the entire surface of the stigma, and afterwards, self-pollination began and continued.

The rates of fruit set in the 10 populations were as follows: controlled hand self-pollination = 93.33%±9.59% (*n* = 30) and cross-pollination = 93.00%±11.19% (*n* = 30) (a mass of pollen was directly placed on the stigma). As regards the seed quantity, the results of self-pollination (16 730±3302 seeds, *n* = 30) and cross-pollination (15 588±3540 seeds, *n* = 30) did not significantly differ from each other (*t* = 0.1239, *P* = 0.4509; *t* = 1.2915, *P* = 0.1008). This result indicated that *P. parishii* was capable of selfing without detrimental effects on the seed set. There was no significant difference (*t* = −1.2141, *P* = 0.1148) between the rates of fruit sets in controlled flowers (84.33%±14.55%, *n* = 30) and in flowers bagged before opening (88.67%±13.06%, *n* = 30). The species was incapable of asexual seed setting, as shown by the emasculated flowers in which no seed was produced (*n* = 30 for each experiment).

## Discussion

To the best of our knowledge, the present study is the first to report such a type of pollination in angiosperms. The flowers were capable of self-fertilization by the automatic regulation of mating location as well as the automatic liquefaction and movement of the anther to span the space between the anther and stigma. This pollination mode differs from others, for example, delayed selfing in *Mimulus* [27], selfing within unopened buds of *Callitriche* [28] and *Corallorhiza odontorhiza* [13], as well as autonomous self-pollination of *Ophrys* aided by gravity and wind [19], of *Paraholcoglossum* via the auto-spinning of the stipe, and of *Chenorchis* aided by ants [9], [11]. No pollinating medium or agent is required, pollination occurs in open flowers, and the process is not a supplemental or back-up mode. The real novelty of this self-fertilization mechanism is that the anthers change to a liquid state. *P. parishii* has floral features adapted to cross-pollination. Its sibling species, *Paphiopedilum dianthum* [16], [17], has flowers similar to those of *P. parishii* (Figure 1F), but instead deceives syrphids into visiting the flower for pollination [29]. Otherwise, *P. dianthum* cannot automatically produce fruits [29] because its anther cannot liquefy and move (Figure 1G). In fact, it does not need to conduct selfing because it grows in a habitat where crossing can occur via pollinating insects [24]. Some self-pollinating mechanisms related to liquefaction have been found. In *Caulokaempferia coenobialis* (Zingiberaceae) [10], pollen grains flowing to the stigma need both oil secretion and a connecting shallow groove on the style to achieve self-pollination. In *Jumellea* and *Eulophia*, spatial separation between the pollinia and stigma hardly exists [25], [26]. In *P. parishii*, there is nothing but a short distance exists between the anther and stigma. To achieve self-pollination, not only the pollen grains but also the anther itself liquefy and wholly step onto the stigma. The self-contained pollination mechanism discussed here is possibly an adaptation to unfavorable environments. Selfing by anther liquefaction and direct stepping onto the stigma from the anthers may have evolved as a strategy for coping with the scarcity of pollinators in the extremely shady and humid (insect-scarce [10]) habitats of *P. parishii* [10], [30], [31]. Given that crossing cannot occur without pollinators, *P. parishii* must evolve a self-breeding strategy for species survival. Owing to the separation of its anther and stigma, and the location of both organs being on a horizontal plane, it cannot place pollen grains on the stigma by gravity as *Cephalanthera grandiflora* [12], which has friable pollen. Its pollen grains conglutinate and become pollinarium with no long stipe, hence the impossibility for pollen grains or pollinarium not be blown to the stigma by wind. There is also no wind because the anther and stigma are both in the cystic labellum and the closing moisture environment is windless. Its stigma lobes and stamen filaments are stubby and cannot move or develop to move the anther and stigma close to each other, such as in the slipper species described by Catling [13]. Pollen grains cannot flow to the stigma with the aid of exudate secretion, such as in *C. coenobialis* [10] and sprouts (e.g., *Eulophia* [12]), from *in situ* because the anther and stigma are both suspended in midair. To overcome the spatial barrier, *P. parishii* rapidly magnifies the anther volume by integral anther liquidation until the anther touches the stigma lobe and totally transfers to the stigma to complete selfing.

Despite the convenience of self-pollination in flowering plants, the detrimental effects of inbreeding that follow repeated selfing have promoted strong natural selection for mating systems, which ensure successful cross-fertilization [2]. On the other hand, crossing is costly and greatly influenced by external conditions. Although inbreeding depression is greatly against the convenience of selfing, selfing still has advantages under specific ecological conditions, such as habitat invasion, unreliable pollination condition, and local population adaptation. A selfing individual can deliver two copied genes to its offspring, whereas crossing can only pass one. By contrast, selfing plants can automatically transfer these advantages [32]. Therefore, the transmutation from crossing superiority to selfing superiority in the evolutionary history of angiosperms occurs periodically [33], [34]. The pollination strategy that is beneficial to the assurance of reproductive success or is less dependent upon pollinator behavior (e.g., self- or anemophilous pollination) is selected to ensure reproduction in environments lacking adequate or reliable pollinators [35]–[37]. Although present-day *P. parishii* carries out self-pollination, its flowers still preserve cross-pollinating characteristics. For example, its pouched lip still acts as a trap for pollinators. This feature is an indication that its automatic self-pollination process must have evolved from entomophilous cross-pollination, and is a result of dwelling in an environment with high humidity and few pollinators. The aphid-like brown spots on its flowers indicate that some primitive forms of *P. parishii* may be mainly pollinated by hoverflies, and these forms must possess some mutational individuals or mutants possessing “delayed selfing.” Delayed selfing is a reproductive assurance mechanism postponing selfing to the point of losing the opportunity for outcrossing. It is beneficial when the pollinator or pollen is limited, and does not increase consumption of what occurs in the outcrossing mechanism [38], [39]. During the years when pollinators fail to visit flowers or are in limited quantities, individuals incapable of selfing may disappear from the population, resulting in an increased the ratio of selfing individuals [38]. This may be an interpretation of the formation of the self-pollination mechanism in *P. parishii*. *P. parishii* has evolved its “anther steps onto the stigma” feature, a peculiar self-pollination mechanism that is an adaptation to environments lacking pollinators. It is a successful evolution indicating that the reproductive assurance of selfing has greater function than inbreeding depression during the evolution of facilitating mating system in plants. We should not underestimate the ability of plants to cope with unfavorable habitats. We should give importance to research on the effects of selfing mechanisms on plant survival.

## Materials and Methods

The flower structure and pollination mechanism of *P. parishii* were observed during the blooming periods (June to September) from 2008 to 2010. The study site was an evergreen broad-leaved forest (1500 m to 1800 m above sea level) in Southern Yunnan, China (22°46′N, 100°36′E).

All necessary permits were obtained for the field studies. The field studies were not located on private lands nor protected areas. The land was controlled by the State Forestry Administration of China. A valid permit was obtained from this authoritative organization. The field tests did not involve collecting nor damaging any plant, animal, or insect. Although *P. parishii* is not a rare or endangered plant, it is under threat and requires protection.

### Observations of floral morphology and flowering phenology

#### Observation of floral morphology

Ten natural populations of *P. parishii* were observed in this study. From each population, more than 10 blooming plants were randomly selected and studied. The following parameters were recorded: number of flowers (buds) in each inflorescence, length of inflorescence, number of inflorescences of each plant, as well as petal length, color, and size.

#### Observation of phenology

The phenological states were also observed and recorded. Ten unbloomed inflorescences from each population were selected and marked. Blooming peculiarities in each single flower and single plant were determined by observing and recording several parameters (i.e., time of opening, closing and corolla fading, morphological changes in lips and petals, number of opening flowers on each inflorescence of each plant every day, as well as opening sequence of the flowers on each inflorescence) every 2 h from 06:00 H to 19:00 H.

#### Observation of pollinium liquefaction

At selected time points, opening flowers of *P. parishii* were collected, dissected (when necessary), and examined under a microscope. The morphologies as well as relative positions of the anthers, pollinia, and stigmas were observed and recorded. Photographs of these floral parts were taken. The pollination process of the flowers in the field sites was also carefully observed and photographed to record the morphology and growth of the flowers, particularly the changes in the anthers and stigmas.

### Observation of the pollination process

Each year from 2008 to 2010, 10 observation sites containing 10 different populations of *P. parishii* were randomly selected. A total of 30 valid sites over 3 years and about 10 flowers at each site were observed. Beginning with the first open flower, the pollination states of each flower during its entire blooming period were observed from 07:00 H to 18:00 H every day until all flowers had opened and undergone the pollination process. A total of 300 flowers were observed for 3 years.

#### Observation of the blooming state

The blooming, pollination, and fruiting states of the flowers in each site were recorded every day until all flowers had opened.

#### Observation of the behaviors of visiting animals

Insects or other animals visiting the flowers of each inflorescence at each site were continuously tracked and photographed. The following were recorded: species of the visitor, number of visits, duration of stay, number of flowers per plant, and number of plants that the same visitor visited. Finally, the insects were caught and preserved as evidence.

#### Measurement of secreted nectar volume and detection of released odor

From 07:00 H to 18:00 H every day, the volume of nectar in the flowers (both unbagged and bagged before blooming) was measured using a 5 µL to 10 µL micropipette. The odor released by the floral lip was detected by careful smelling.

#### Observation and quantification of fruit set and seeds

A successful fruit set following pollination is easily identified by the morphology of non-fallen floral organs and fruit enlargement. The rate of the natural fruit set was determined based on the observation data from the 300 flowers. Each year, 10 capsules were randomly selected and the seeds from each capsule were observed and counted under a microscope.

### Test of the breeding system

#### Experiments on manual self- and cross-pollination

A total of 30 sites (15 pairs) were randomly selected for controlled manual self-pollination and manual cross-pollination from 2008 to 2010 (10 sites, five pairs each year). Ten flowers were observed at each site.

#### Manual self-pollination

The flower was bagged before blooming. After blooming but before fertilization, the bag was temporarily opened. The pollinia of the flower were peeled off and placed onto its own stigma. The flower was bagged again. Changes in the flower and fruit set state were recorded.

#### Manual cross-pollination

Flowers from the paired plants at the same site were bagged before blooming. After blooming but before fertilization, the bags were temporarily opened. The pollinia of one flower were peeled off and placed onto the stigma of a different flower of another plant, and vice versa. The flowers were bagged again. Changes in the flower and fruit set state were recorded.

#### Experiments on natural and bagged self-pollination

A total of 30 sites (15 pairs) were randomly selected for controlled natural self-pollination and bagged self-pollination from 2008 to 2010 (10 sites, five pairs each year). Ten flowers were observed in each site. For natural self-pollination, the flower was not manipulated. For bagged self-pollination, the soon-to-bloom flower was covered with a transparent bag to prevent insect entry. The pollination and fruit set states of both samples were observed and recorded.

#### Natural self-pollination in the laboratory

A total of 15 plants (5 plants each year) were randomly selected and transplanted to our laboratory (80% to 95% relative humidity) before blooming. All plants were untreated. The states of pollination were observed and recorded.

#### Experiments on bagged asexual reproduction

A total of 18 (nine pairs) controlled sites (six sites, three pairs each year) were randomly selected. Ten flowers were observed in each site, and all were bagged before blooming. After blooming but before fertilization, the pollinia of each flower in both paired sites were peeled off and discarded. The flowers in one site were bagged, and those in the other site were left untreated. The pollination and fruit set states were observed and recorded.

### Experiment on restricted flower opening

A total of 18 sites were randomly selected for examining restricted flower opening. Young inflorescences were enclosed in a small transparent bag to restrict natural flower opening. There were six sites each year and two inflorescences in each site. The pollination and fruit set states were observed and recorded.
